# Exploring the perceptions of oncology healthcare professionals on introducing home-based palliative care for patients with advanced cancer in Gaza - a qualitative study

**DOI:** 10.3389/fonc.2026.1786947

**Published:** 2026-03-12

**Authors:** Haytham Abusenjar, Fatima Mansour, Tracy Daou, Khamis Elessi, Rana Yamout

**Affiliations:** 1 Global Health Institute, American University of Beirut, Beirut, Lebanon; 2 Faculty of Medicine, Islamic University of Gaza, Gaza, Palestine; 3 Internal Medicine, American University of Beirut Medical Center, Beirut, Lebanon

**Keywords:** advanced cancer, conflict-affected settings, Gaza, home-based palliative care, humanitarian settings, oncology healthcare professionals, palliative care

## Abstract

**Introduction:**

Palliative care aims to improve the quality of life for those with life-limiting diseases. It is still an emerging specialty in Palestine with limited governmental funding and underdeveloped national palliative care policies. Yet, the high burden of cancer and ongoing conflict in Gaza have increased the need for palliative care. Home-based palliative care has emerged as a promising approach to address the unmet needs of patients with advanced cancer, particularly in low-resource settings. This research aims to explore the perceptions of the oncology healthcare team on introducing home-based palliative care for patients with advanced cancer in a tertiary center in Gaza.

**Methods:**

This is a qualitative study that was carried out at the Turkish Palestinian Friendship Hospital in Gaza. Semi structured face-to-face interviews were conducted with 15 healthcare professionals working in the oncology department. Data collection took place between March and May 2023, and the data were analyzed via a thematic analysis approach to identify themes and subthemes.

**Results:**

The thematic analysis generated four main themes and fourteen subthemes were generated from the thematic analysis. The main themes include general perceptions about palliative care, perceptions of home-based palliative care, challenges in the provision of home-based palliative care, and suggestions to overcome challenges. The purpose of palliative care was recognized to extend beyond standard physical care. The introduction of palliative care services in Gaza remains in its infancy. Shortages of palliative care experts, budget constraints, endemic conflicts and cultural sensitivity were reported to be significant challenges for its development. However, several suggestions to address these obstacles have also been presented.

**Discussion:**

Although developing home-based palliative care could be beneficial to cancer patients, their families and healthcare system, the political conflict represents one of the main barriers to its development. To comprehensively understand and develop an effective model for home-based palliative care, future research should include broader perspectives including families and communities.

## 1 Background

Cancer is the second major cause of mortality in Palestine after cardiovascular disease, accounting for approximately 10.9% of total deaths in 2021 in the Gaza Strip (1). Most cancer patients are diagnosed at later stages of the disease, making treatment more challenging and often resulting in poorer health-related quality of life as well as a greater economic burden on patients, their families, and the healthcare system (1, 2).

Structurally, the Palestinian Ministry of Health is the main provider of secondary and tertiary healthcare services in Gaza, with contributions from UNRWA and other NGOs (3). There are large gaps in healthcare services provided in Gaza, related primarily to the ongoing political conflict. For example, the majority of Gazan patients are compelled to seek treatment abroad due to a lack of treatments and diagnostic measures, especially PET scans. However, frequent blockades, border closures, and restrictions imposed by Israeli authorities often lead to delays or denials of travel permits. These barriers result in premature death and unnecessary suffering for many patients (3).

PC is not officially integrated into the healthcare system as seen in other conflict countries in the region such as Iraq, Syria, and Afghanistan (4); however, the Turkish Palestinian Friendship Hospital (TPFH) is the main provider in Gaza (5–7). The first multidisciplinary palliative care team was established at the TPFH in 2022 in collaboration with Cairdeas International Palliative Care Trust (5). The team consists of a number of healthcare professionals from diverse backgrounds, including pain management physicians, psychologists, nurses, physiotherapists, pharmacists and dieticians. They conduct a daily morning round on all inpatients’ cancer cases to identify those who need palliation most. Nonetheless, border closures limit the import of essential medical supplies and equipment, creating additional barriers to providing effective palliative care in Gaza. Furthermore, power outages and a lack of skilled healthcare personnel further compound the difficulties in delivering adequate care in such a resource-constrained and conflict-affected environment as seen in other countries in the regions (8).

Home-based palliative care (HBPC) could be one option to mitigate suffering in a conflict-impacted place where reaching hospitals is very challenging (9–11). Accordingly, home-based palliative care (HBPC) presents a viable solution to mitigate suffering in conflict affected settings where access to hospitals becomes extremely challenging. Research has shown that HBPC is effective in improving quality of life, reducing hospital admissions, and reducing hospitalization costs (12, 13).

To our knowledge, limited research has focused on assessing palliative care services in Gaza. Previous relevant studies have mainly covered cancer epidemiology, and have not explored the views and needs of the relevant broader community services (5, 11). This qualitative exploration aims to fill a research gap by investigating the perception of the oncology healthcare team in a tertiary center in Gaza regarding the introduction of HBPC for patients with advanced cancer.

## 2 Methods

### 2.1 Study design

This qualitative study targeted the oncology healthcare team working at the TPFH and was conducted between March and May 2023. Purposive sampling was chosen to ensure the inclusion of participants with direct experience in oncology and palliative care, representing diverse professional backgrounds. Data collection was discontinued upon reaching thematic saturation, and successive interviews yielded no new codes or themes and further data collection was deemed unlikely to generate additional insights relevant to the study objectives.

### 2.2 Study subjects

Expert purposive sampling was employed to select participants who were knowledgeable and experienced care providers. The cohort comprised 15 individuals (4 nurses, 1 pharmacist, 2 physiotherapists, 1 psychologist, 2 residents, and 5 physicians including 2 hematologists and 3 solid tumor oncologists). The inclusion criteria were being involved in providing direct palliative care for patients with advanced cancer at the hospital and willingness to participate in the study. The participants were all males selected from multidisciplinary backgrounds to ensure maximum representation.

### 2.3 Data collection

Data were collected via semi structured face-to-face interviews between March and May 2023. It is important to note that data collection for this study was conducted prior to the war in Gaza in October 2023. Since then, the humanitarian situation has significantly changed, which may affect the applicability of some findings or interpretations presented in this manuscript. In total, 15 individual interviews were conducted in person. An audio recorder was used while data were collected after consent was obtained from the participants, and the questions were administered in Arabic. The duration of each interview was between 15 and 30 minutes. The interview guide was developed by the research team on the basis of the literature. Data collection continued until data saturation was reached. The detailed interview guide is included in the **Supplementary Material** “See **Supplementary Interview Guide**”.

### 2.4 Analysis

The data analysis was conducted simultaneously with the interviews using thematic analysis approach proposed by (14). The audio-recorded data were transcribed verbatim and then analyzed via thematic analysis. An inductive approach was used to guide the analysis, whereby the transcripts were independently reviewed by researchers to identify emerging codes. The codes that emerged from the transcripts were categorized into themes and subthemes.

## 3 Ethical considerations

The investigation received ethical approval from the Helsinki Committee in Gaza and administrative approval from the Ministry of Health to proceed with the research at the designated hospital. Further approval from the Institutional Review Board (IRB) at the American University of Beirut was also obtained. Prior to conducting the interviews, the study objectives were thoroughly communicated to all the participants, ensuring transparency. Participants’ anonymity and confidentiality were strictly maintained, with the assurance of their right to withdraw from the research at any stage. Written informed consent was obtained from each participant. The audio recordings and transcriptions were securely stored on a password-protected computer, and only the research team had access to these data.

## 4 Results

In this study, the data were collected through 15 face–to-face interviews. The participants’ characteristics are detailed in **Supplementary Table S1**, which can be found in the **Supplementary Materials**.

Below are the main four themes that emerged: A detailed table of the thematic analysis can be found in the **Supplementary Material** “See **Supplementary Table 2**”.

### 4.1 General perceptions about palliative care

#### 4.1.1 Acknowledgement of the palliative care definition

The participants described palliative care as a holistic approach that addresses the physical, emotional, social, and spiritual needs of patients with advanced illnesses. Its complexity varies on the basis of factors such as disease stage and patient condition. However, the depth of understanding differed across professional categories. Physicians demonstrated a more comprehensive and nuanced understanding of palliative care, often articulating its multidimensional scope and role across the disease trajectory. In contrast, some non-physician participants described palliative care in more limited terms, focusing primarily on symptom management.

“Palliative care is a comprehensive approach that covers various services, including physical, psychosocial and spiritual services.”

#### 4.1.2 Misconceptions and negative perceptions toward palliative care

Several misconceptions about palliative care were identified among participants, including the belief that palliative care is solely limited to cancer patients. Additionally, the confusion between palliative care and hospice care often leads to the misunderstanding that palliative care is delivered only at the final stages of a disease.

“Patients’ families believe that receiving palliative care indicates that the patient is nearing death. This association creates a negative perception of palliative care.”

#### 4.1.3 Shifting perceptions and overcoming stigma in cancer and palliative care

While cancer and home-based care face stigma in Gaza, increasing cancer rates are driving greater acceptance of palliative care. Promoting home-based care within an Islamic framework, which values family caregiving, may help overcome this stigma.

“The more we talk about cancer and palliative care, the more we break the stigma.”

### 4.2 Perceptions of the benefits of home-based palliative care

#### 4.2.1 Benefits to the patient

The participants believed that HBPCs provide better overall care than hospitals do. It improves patients’ emotional well-being, lowers infection risk, reduces family costs, offers greater convenience, especially for those with limited mobility, and minimizes waiting times for dispensing medication.

“HBPC would save many patients the hassle of coming to the hospital and waiting in long queues for medications to be dispensed or to be seen by a doctor. These services will reach the patient while he is at home instead.”

#### 4.2.2 Benefits to the healthcare system

The participants agreed that HBPC can ease the burden on the local healthcare system by reducing hospitalization costs and bed occupancy, allowing resources to be redirected to patients with greater needs.

“HBPC frees up hospital beds that can be allocated to other patients in need, optimizing the utilization of healthcare facilities.”

### 4.3 Barriers to HBPC implementation

#### 4.3.1 Geopolitical context

Discrepancies in opinions among participants emerged regarding the impact of political instability on the implementation of HBPC, with some perceiving the ongoing state of emergency as a hindrance.

Others have asserted that healthcare workers have adapted to address these limitations. Nonetheless, all agreed that Gaza’s conflict-affected status and geography present significant barriers to the development of palliative care. The participants expressed that cancer patients encounter notable difficulties accessing hospital-based care during periods of conflict, worsening their suffering. They also articulated that in such times, the focus of care shifts to life-saving measures, impacting services provided to patients requiring palliative care.

“There may be challenges that hinder the implementation of home palliative care, especially in Gaza, because it is a conflict affected area, and it is different from the rest of the world.”

#### 4.3.2 Limited resources

Most participants agreed that the scarcity of resources, including financial constraints and restrictions imposed on certain medicines or medical devices from entering Gaza, profoundly affects the implementation of palliative care services both within hospitals and in home settings.

“It is possible that there is a ban on some medicines or devices, as it affects the provision of services in the hospital and at home.”

Participants further emphasized that limited governmental support and low prioritization of palliative care exacerbate these resource constraints. Although some progress has been achieved through collaborations with international organizations and academic institutions, such as the Islamic University, services remain largely hospital-based. Expanding community- and home-based palliative care was viewed as crucial but requires government approval and a clear strategy for development. In the absence of a formalized system, informal palliative care services, whether hospital- or home-based, are often provided by families, non-governmental organizations, and volunteers. However, participants noted that these efforts do not ensure comprehensive coverage of all palliative care domains, particularly psychological, social, and spiritual support. One participant described the emergence of the palliative care initiative:

“It is an initiative that I took in 2010 after the death of my father, who died in pain inside a hospital. After this incident, I made the decision that this pain should not be felt by the patient before his death, so I began an initiative to spread the culture of comprehensive pain medicine and palliative care as a new concept in medicine in the Gaza Strip.”

Furthermore, the respondents highlighted the economic situation as a significant barrier to HBPC straining the healthcare system and patients’ families, particularly those who are already poor. Shortages of funds, medical supplies, trained personnel, and essential equipment limit the ability of the Ministry of Health to provide home-based care because of its high costs and resource needs. They also reported that funding gaps and a lack adequate support lead to team burnout. Moreover, inadequate pain management skills, insufficient medical equipment and frequent power outages often necessitate transferring patients to better-equipped hospitals.

“It is difficult to care for the patient at home because essential resources, such as electricity, are often unreliable, whereas hospitals have these facilities available at all times.”

Moreover, a dearth of palliative care expertise and relevant training in Gaza exert paramount strains on the existing healthcare team, leading to burnout and prompting medical staff to consider alternative career paths, as claimed by the participants. The paucity of specialized knowledge and skills not only inhibits effective pain management, but also highlights challenges in maintaining continuous follow-up for patients.

“From my point of view, the most prominent challenge is continuity of care because the team dedicated to provide home care is not the same team that works at the hospital. The home team must be free throughout the week and have a day meeting at the hospital to update the care plan for patients in cooperation with the consultant oncologist at the hospital.”

#### 4.3.4 Cultural considerations

Societal norms and gender roles significantly influence palliative care delivery in Gaza. For example, male caregivers are typically assigned to male patients and vice versa. Owing to public resistance to women providing home care and traditional standards, female medical staff face challenges that limit their role. Similarly, families may refuse male providers for female patients at home. One participant noted that societal norms often confine women to specific care settings, reducing opportunities for them to actively engage in broader healthcare delivery initiatives such as HBPC. This restriction reflects the broader societal barriers that women face in Gaza, which hinders their ability to contribute fully to professional caregiving roles outside hospital settings.

“Gaza being an Eastern society with a Muslim majority, it is considered inappropriate for a male doctor to examine a female patient and vice versa.”

Moreover, some participants considered hospitals to be the optimal location for patient treatment in Gaza. Therefore, shifting the perception from providing care at the hospital to providing care at home might be challenging and might be refused by patients and their families.

“People are accustomed to the hospital being the ideal place for treatment.”

### 4.4 Strategies to advance palliative care in Gaza

#### 4.4.1 Policy integration, collaborations, and research

To implement sustainable palliative care programs effectively in Gaza, participants recommended policy changes, integrating services into healthcare units, collaborating with international organizations for resources and support, and, most importantly, generating evidence to demonstrate the effectiveness of palliative care to officials.

“I believe that scientific research is the greatest guide and motivator to convince administration with a specific project.”

#### 4.4.2 Developing a continuum of care model

The participants recommended key strategies for enhancing palliative care in Gaza, including a system ensuring smooth transfer of patients from hospitals to home-based care, access to specialized and well-equipped home care centers, a dedicated team for regular home visits and ongoing patient support, and the use of telehealth to improve communication and coordination.

“The institution has better resources and can create a structured program for regular patient visits and communication, including a phone app or video chat for patient inquiries, especially useful during conflicts.”

#### 4.4.3 Workforce development and institutional support

The participants identified several support mechanisms to enhance the well-being of palliative care providers in Gaza, including financial, psychological, social, and academic support, along with incentives and improved working conditions. They suggested reducing workload through allowances and extended leave to combat burnout and proposed establishing dedicated training institutes to enhance healthcare professionals’ skills and knowledge.

“Carrying out training courses specialized in palliative care to develop a medical team specialized in this field, whether by holding internal or other training courses outside Gaza in advanced palliative care centers”

Furthermore, the participants emphasized the importance of raising awareness about palliative care through targeted promotion, starting with educating medical personnel and stakeholders. Securing stable funding for human resources and recruitment is also vital to ensure the sustainability and growth of HBPC units, allowing for full-time staff and adequate resources. Insufficient financial support could hinder the long-term development of palliative care services in Gaza.

“Awareness first begins with a broad awareness campaign that begins with medical personnel before the general public.”

#### 4.4.4 Family and caregivers’ engagement

Participants highlighted the importance of involving and educating family members about providing palliative care at home and establishing strong communication channels between the care team, patient, and family. Patients’ economic situations and caregivers’ knowledge should also be taken into consideration when patients are discharged for HBPC. Patient education is also crucial for addressing misconceptions about pain medication and promoting awareness of HBPC.

“Increasing awareness about HBPCs will increase acceptance among patients and their families.”

#### 4.4.5 Cultural sensitivity and inclusivity in palliative care

The participants contributed to the idea of respecting diversity and cultural differences in care delivery in different ways. First, the palliative care team should prioritize respecting the confidentiality of patients and demonstrating sensitivity to patients’ religious beliefs. In addition, palliative care should be inclusive and accessible to people from all backgrounds. Finally, medical staff must provide care to patients in line with their cultural and religious values.

“A holistic approach is vital for meeting patients’ needs, especially in a Muslim population, with specialists who can convey ideas of reward and affliction in line with their cultural and religious beliefs.”

## 5 Discussion

This study explored the perceptions of oncology healthcare professionals regarding the introduction of home-based palliative care (HBPC) for patients with advanced cancer in Gaza. The study included 15 participants, all male, with diverse professional backgrounds, including oncologists, nurses, physiotherapists, hematologists, a pharmacist, a psychologist, and medical residents. Their years of experience varied, with some having less than five years in palliative care and others having more than two decades of experience in oncology and palliative care.

Four main themes emerged from the analysis, each containing several subthemes that provide a deeper understanding of the potential benefits and barriers associated with HBPC. The first theme, “*General Perceptions about Palliative Care*,” highlighted the participants’ understanding of palliative care as a holistic approach addressing physical, social, and spiritual needs. However, misconceptions among participants were prevalent, with some linking palliative care solely with end-of-life services. The second theme, “*perceptions of home-based palliative care,”* revealed that HBPC was viewed favorably for its ability to improve patients’ quality of life, reduce hospital admissions, and ease financial burdens on families. The third theme, “*Challenges in the Provision of HBPCs*,” outlined the significant barriers to implementation, including geopolitical instability, limited resources, shortages of trained personnel, and cultural factors influencing the acceptability of HBPCs. The final theme, “*Suggestions to Overcome Challenges*,” focused on policy recommendations, capacity-building efforts, and community engagement strategies to facilitate the integration of HBPCs into the existing healthcare system.

While previous relevant studies in Gaza have focused primarily on cancer epidemiology, this research uniquely investigates the perspectives of healthcare professionals on the feasibility and implementation of HBPC (5, 11). This study aligns with the international literature emphasizing the importance of a multidisciplinary approach and the need for strong community support in developing effective palliative care programs (5, 7). The findings align with those of global and regional studies that emphasize the holistic nature of palliative care, addressing physical, psychosocial, and spiritual needs. This finding is also in line with those of previous studies that explored palliative care perceptions and challenges in other similar resource-limited settings. For example, the misconceptions and negative perceptions toward palliative care identified in our study echo similar findings from the region and other regions where palliative care is still emerging, such as sub-Saharan Africa and parts of South Asia (10, 15–18). The WHO reports that a lack of awareness and widespread misconceptions about the role of palliative care significantly impedes its integration into health systems worldwide. In Gaza, this challenge is compounded by cultural stigma and limited public education. Addressing these barriers will require targeted awareness campaigns and educational initiatives, similar to those recommended by the WHO for advancing palliative care integration in low-resource settings(WHO, n.d.). Home-based care, driven by cultural norms, particularly faces stigma in Gaza. This highlights the deeply rooted societal fear surrounding illness and health. Nevertheless, such stigma mirrors findings from other Muslim-majority contexts, where linking caregiving to religious and family responsibilities has been suggested to encourage acceptance of HBPC (12, 19)Similarly, the benefits of HBPC reported by our participants are consistent with the international literature on HBPC effectiveness. For example, being in a comfort zone at home allows patients to experience a sense of normalcy and security, which can significantly improve their mental and emotional state, as claimed by other studies (13, 20) Another important benefit highlighted by respondents was the lower risk of infection in those receiving care at home than in those receiving care at hospitals, and this benefit is well documented in research by Alizadeh et al. (21). Our findings also emphasized the convenience and reduced financial burden for both patients and families in Gaza, where the vast majority of people are poor (22). The HBPC can significantly reduce these costs by avoiding hospital admission fees, transportation expenses, and potential losses of income for caregivers (23).

Given the overwhelmed local healthcare system, the study outcomes suggested that introducing the HBPC could reduce the workload on healthcare services, allowing finite assets to be shifted toward the most urgent needs. Other studies support this view, showing that HBPC contributes to reducing hospital admissions, freeing more beds, and reducing hospitalization costs (23–25).

While some challenges with palliative care are common internationally, the unique situation in Gaza is deeply intertwined with the ongoing political conflict and its impact on the healthcare system and wider society (3, 11).

Gaza’s geopolitical context represents a major barrier to the provision of HBPCs in Gaza, according to the participants. Ongoing conflict significantly affects healthcare delivery. For example, security threats and the potential for violence can disrupt services and deter healthcare workers from providing home visits to patients, which might exacerbate their suffering. It often shifts focus to emergency and life-saving measures at the expense of palliative care. Moreover, border closures and restrictions on importing medical supplies and equipment create severe barriers to providing effective palliative care. Additionally, the frequent state of emergency places immense pressure on the healthcare system, making it challenging to allocate resources to new palliative care services. Shortage of skilled personnel, particularly in specialized palliative care areas, is another challenge. Importantly, the support mechanisms proposed by participants should not be interpreted solely as efforts to improve individual provider well-being. Rather, they reflect a broader call for the formal recognition and institutionalization of palliative care as a valued medical specialty in Gaza. Financial incentives, improved working conditions, and access to structured training targeting different disciplines are framed as elements of a viable and rewarding career pathway, aimed at attracting and retaining professionals in the field. However, such a dire context necessitates innovative strategies to develop palliative care services that can operate effectively within these limitations.

An important limitation of this study is that all participants were male, which may have shaped the perspectives captured. Gender roles influence professional experiences, caregiving expectations, communication with patients and families, and perceptions of safety and mobility in home-based care, particularly in conflict-affected settings such as Gaza. The absence of female healthcare professionals may therefore limit the generalizability of the findings and the exploration of gender-specific insights related to psychosocial care and household-based service delivery. Future research should intentionally include female providers to enable a more comprehensive and gender-responsive understanding of HBPC.

The study highlights several potential solutions. First, the development of a robust national palliative care policy, including clear guidelines for HBPC implementation within the existing healthcare system, is crucial and could be generated through evidence-based research. Second, contingency plans should be developed, and alternative service delivery models should be explored. This may include the use of telemedicine, the establishment of mobile clinics, and strengthening of community-based support networks. In parallel, targeted capacity-building initiatives are needed to equip care providers with the competencies required to deliver home-based palliative care. Third, building a dedicated team that is separate from hospital-based staff could improve the quality and consistency of care. Finally, investing in strong community partnerships, including religious leaders, community organizations, and patient advocacy groups, would be invaluable for HBPC introduction in terms of raising awareness, addressing cultural sensitivities, and gaining community support for HBPC.

## 6 Limitations

The study is limited to a single hospital and has a small sample size, which may affect the generalizability of the findings. The sample’s homogeneity (all males) presents an additional constraint. The research was conducted between March and May 2023, which may have been a period of relative stability in Gaza. The perceptions and challenges reported might differ significantly, especially after events following October 7, 2023.

## 7 Conclusion

This study found that while introducing HBPC services could be beneficial to cancer patients, their families and the healthcare system in Gaza, yet geopolitical instability, finite resources, and cultural barriers represent major barriers to their development.

Based on findings, the study recommends the need for policy reforms, stable funding, workforce development, and community engagement to support sustainable local HBPC services. Future research should incorporate broader perspectives, including families to inform a comprehensive and culturally sensitive HBPC model. Moreover, a coordinated effort at both local, regional, and international levels is essential for making the HBPC more accessible and effective in Gaza and in countries with similar contexts.

## Data Availability

The original contributions presented in the study are included in the article/**Supplementary Material**. Further inquiries can be directed to the corresponding author.

## References

[1] Halahleh K Abu-Rmeileh NME . General oncology care in Palestine. In: Al-Shamsi HO Abu-Gheida IH Iqbal F Al-Awadhi A , editors. Cancer in the arab world. Singapore: Springer (2022). doi: 10.1007/978-981-16-7945-2, PMID:

[2] Mahdi H Mula-Hussain L Ramzi ZS Tolba M Abdel-Rahman O Abu-Gheida I . Cancer burden among Arab-world females in 2020: working toward improving outcomes. JCO Global Oncol. (2022) 8:e2100415. doi: 10.1200/GO.21.00415, PMID: 35259001 PMC8920429

[3] Giacaman R Khatib R Shabaneh L Ramlawi A Sabri B Sabatinelli G . Health status and health services in the occupied Palestinian territory. Lancet. (2009) 373:837–49. doi: 10.1016/S0140-6736(09)60107-0, PMID: 40881048

[4] Mohsin K Mula-Hussain L Gilson R . HealthCare Access Barrier (HCAB) framework for the barriers to cancer care during conflicts: perspective from Iraq. BMJ Oncol. (2024) 3:e000252. doi: 10.1136/bmjonc-2023-000252, PMID: 39886185 PMC11203082

[5] Abu-Odah H Molassiotis A Liu JYW . Gathering policymakers’ perspectives as an essential step in planning and implementing palliative care services at a national level: an example from a resource-limited country. BMC Palliative Care. (2022) 21:43. doi: 10.1186/s12904-022-00936-1, PMID: 35354398 PMC8967559

[6] Graneheim UH Lundman B . Qualitative content analysis in nursing research: concepts, procedures and measures to achieve trustworthiness. Nurse Educ Today. (2004) 24:105–12. doi: 10.1016/j.nedt.2003.10.001, PMID: 14769454

[7] Hughes MC Vernon E Hainstock A . The effectiveness of community-based palliative care programme components: a systematic review. Age Ageing. (2023) 52:afad174. doi: 10.1093/ageing/afad175, PMID: 37740895 PMC10517647

[8] Mula-Hussain L Mahdi H Ramzi ZS Tolba M Zaghloul MS Benbrahim Z . Cancer burden among Arab world males in 2020: The need for a better approach to improve outcome. JCO Global Oncol. (2022) 8:e2100407. doi: 10.1200/GO.21.00407, PMID: 35353549 PMC9005253

[9] Hamad BA Skaik N Abu-Odah H . Evaluation of palliative care services provided to cancer patients in the gaza strip. J US-China Med Sci. (2016) 13:95–107. doi: 10.17265/1548-6648/2016.02.006

[10] Halahleh K . Cancer in the arab world. In: Cancer in the arab world. Singapore: Springer Singapore (2022). doi: 10.1007/978-981-16-7945-2, PMID:

[11] Coghlan R . *Caring for the seriously ill and dying in humanitarian crisis and conflict: Shifting a ‘usual’ palliative care paradigm to Gaza’s own* [PhD Thesis]. Deakin University (2023).

[12] Cox S Gazzard B . Models for palliative care in sub-Saharan Africa. Int Health. (2010) 2:77–8. doi: 10.1016/j.inhe.2010.04.003, PMID: 24037466

[13] Meier DE Brawley OW . Palliative care and the quality of life. J Clin Oncol. (2011) 29:2750–2. doi: 10.1200/JCO.2011.35.9729, PMID: 21670456 PMC3139393

[14] Braun V Clarke V . Using thematic analysis in psychology. Qual Res Psychol. (2006) 3:77–101. doi: 10.1191/1478088706qp063oa, PMID: 32100154

[15] Gysels M Pell C Straus L Pool R . End of life care in sub-Saharan Africa: a systematic review of the qualitative literature. BMC Palliative Care. (2011) 10:6. doi: 10.1186/1472-684X-10-6, PMID: 21388538 PMC3070681

[16] Silbermann M Jaloudi MA . Setting a higher standard in cancer care: Isn’t it time to bring about a change in cancer palliation. J Palliat Care Med. (2014) 4:e128. doi: 10.4172/2165-7386.1000e128, PMID: 39887974

[17] Weng XR Nakdali R Almoosawi B Al Saeed M Maiser S Al Banna M . Health care providers’ attitudes and beliefs on providing palliative care to patients in Bahrain: Findings from a qualitative study. J Pain Symptom Manage. (2021) 62:98–106. doi: 10.1016/j.jpainsymman.2020.11.006, PMID: 33188863

[18] WHO . Palliative care. Available online at: https://www.who.int/news-room/fact-sheets/detail/palliative-care (Accessed May 23, 2025).

[19] Shen MJ Wellman JD . Evidence of palliative care stigma: The role of negative stereotypes in preventing willingness to use palliative care. Palliative Supportive Care. (2019) 17:374–80. doi: 10.1017/S1478951518000834, PMID: 30520405 PMC6551309

[20] Giordano V Guillari A Lanzuise A Virgolesi M Di Vaio L Rea T . EFFECTIVENESS OF PALLIATIVE HOME CARE ON CLINICAL, EMOTIONAL AND ECONOMIC WELL-BEING AT THE END OF LIFE: A NARRATIVE REVIEW. Nsc Nurs. (2024) 4:27–45. doi: 10.32549/OPI-NSC-111

[21] Alizadeh Z Rohani C Rassouli M Ilkhani M Hazrati M . Challenges of integrated home-based palliative care services for cancer patients during the COVID-19 pandemic: A qualitative content analysis. Home Health Care Manage Pract. (2023) 35:180–9. doi: 10.1177/10848223221134780, PMID: 38603240 PMC9672982

[22] World Bank . World bank issues new update on the palestinian economy (2024). Available online at: https://www.worldbank.org/en/news/press-release/2024/05/23/world-bank-issues-new-update-on-the-palestinian-economy (Accessed May 23, 2025).

[23] Gonzalez-Jaramillo V Fuhrer V Gonzalez-Jaramillo N Kopp-Heim D Eychmüller S Maessen M . Impact of home-based palliative care on health care costs and hospital use: A systematic review. Palliative Supportive Care. (2021) 19:474–87. doi: 10.1017/S1478951520001315, PMID: 33295269

[24] Iupati S Stanley J Egan R MacLeod R Davies C Spence H . Systematic review of models of effective community specialist palliative care services for evidence of improved patient-related outcomes, equity, integration, and health service utilization. J Palliative Med. (2023) 26:1562–77. doi: 10.1089/jpm.2022.0461, PMID: 37366688

[25] Roberts B Robertson M Ojukwu EI Wu DS . Home based palliative care: known benefits and future directions. Curr Geriatrics Rep. (2021) 10:141–7. doi: 10.1007/s13670-021-00372-8, PMID: 34849331 PMC8614075

