# Supplementary material for: Exploring the perceptions of oncology healthcare professionals on introducing home-based palliative care for patients with advanced cancer in Gaza - a qualitative study
Source: Front Oncol. 2026 Mar 12;16:1786947. doi: 10.3389/fonc.2026.1786947 (PMC13017347; doi:10.3389/fonc.2026.1786947)
Supplement: Supplementary file 1 [file Table1.docx]

**Exploring the Perceptions of the Oncology Healthcare Team on Introducing Home-Based Palliative Care for Patients with Advanced Cancer in Gaza**

| **Domain** | **Questions for healthcare professionals (oncologists and hematologists)** |
| --- | --- |
| **General knowledge of palliative care** | 1. Describe what does palliative care means to you? |
| **Perceptions of home-based palliative care** | 1. How would applying home-based palliative care be beneficial for cancer patients in Gaza?  2.a. What are some challenges that can prevent the implementation of home-based palliative care in conflict settings like Gaza on a:  - political level (note to data collector: probe on ability to attain an exit permit)  - practical level (note to data collector: probe on availability of medical resources, medical providers, and policies set)  -Others (note to data collector: probe on misconceptions)  2.b. How do you think these challenges could be overcome?  3.a. What are the barriers that healthcare providers may experience while delivering home-based palliative care?  3.b. How do you think these barriers could be overcome?  4.a. How would introducing home-based palliative care impact the Palestinian healthcare system in terms of using health resources?  4.b. How would introducing home-based palliative care impact the Palestinian healthcare system in terms of hospitalization costs?  4.c. How would introducing home-based palliative care impact the Palestinian healthcare system in terms of number of hospital beds available?  5. When taking into consideration the shortage of a well-trained workforce in conflict settings, in your opinion, what would be the proper way to deliver home-based palliative care?  6. What are the criteria that should be considered when you want to refer your patients to receive palliative care at home in the future?  7. Can you tell me about any cultural considerations that are important to consider while delivering home-based palliative care?  8. If a cancer center developed a program to send a community health worker to visit patients at home to help coordinate their healthcare, how interested do you think patients would be in this type of program?  9.To your knowledge, are there any informal palliative care services being provided?  a. at home (yes/no)  b. in the hospital (yes/no)  Please elaborate. |
| **Other** | 1. Is there anything else you would like to share with us regarding introducing home-based palliative care to cancer patients in Gaza? |

**استكشاف تصورات فريق طب الأورام للرعاية الصحية حول تقديم الرعاية التلطيفية المنزلية** **لمرضى السرطان المتقدم في غزة**

| **أسئلة لمتخصصي الرعاية الصحية (أطباء الأورام وأخصائي أمراض الدم)** | **النطاق** |
| --- | --- |
| صف لي ماذا تعني لك الرعاية التلطيفية؟ | المعرفة العامة حول الرعاية التلطيفية |
| .1 كيف سيكون تطبيق الرعاية التلطيفية المنزلية مفيدا لمرضى السرطان في غزة  .2.a ما هي بعض التحديات التي يمكن ان تمنع/تعيق تطبيق الرعاية التلطيفية المنزلية في غزة باعتبارها منطقة متأثرة بالصراع على:  - المستوى السياسي (ملاحظة لجامع البيانات: فحص القدرة على الحصول على تصريح خروج)؟  - المستوى العملي (ملاحظة لمجمع البيانات: التحقيق في مدى توفر الموارد الطبية ومقدمي الخدمات الطبية والسياسات المحددة)  أخرى (ملاحظة لمجمع البيانات: التحقيق في المفاهيم الخاطئة)  2.b.كيف برأيك يمكن التغلب على هذه التحديات؟  3.a. ما هي المعيقات التي من الممكن ان تواجه مزودي الرعاية الصحية عند تقديم الرعاية التلطيفية المنزلية؟  3.b. كيف برأيك يمكن التغلب على هذه المعيقات؟  4.a.كيف سيؤثر ادخال الرعاية التلطيفية المنزلية على النظام الصحي الفلسطيني من حيث استخدام الموارد؟  4.b.كيف سيؤثر ادخال الرعاية التلطيفية المنزلية على النظام الصحي الفلسطيني من حيث تكاليف المبيت/العلاج في المستشفى؟  4.c. كيف سيؤثر ادخال الرعاية التلطيفية المنزلية على النظام الصحي الفلسطيني من حيث عدد الأسرة المتاحة في المستشفيات؟  5. بالأخذ في الحسبان النقص في الأيدي العاملة المدربة جيدا في أماكن النزاع, برأيك، ما هي الطريقة المناسبة/الملائمة لتقديم الرعاية التلطيفية المنزلية؟   6.ما هي المعايير التي يجب ان تؤخذ بالحسبان عندما تريد تحويل مريضك لتلقي الرعاية التلطيفية في المنزل في المستقبل؟  7. هل من الممكن ان تخبرنا عن اية اعتبارات ثقافية من المهم اخذها بعين الاعتبار عند تقديم الرعاية التلطيفية المنزلية؟   8.لنفترض مثلا ان مركز سرطان ما قد طور برنامج لإرسال عامل رعاية صحية مجتمعية لزيارة المرضى في منازلهم للمساعدة في تنسيق الرعاية الصحية المقدمة لديهم، ما مدى اهتمام/استجابة المرضى لهذا النوع من البرامج/الرعاية برأيك؟  9- بناءً على معرفتك، هل هناك أي تقديم لخدمات الرعاية التلطيفية غير الرسمية؟ من فضلك وضح.  أ. في البيت (نعم/لا)  ب. في المستشفى (نعم/لا)  من فضلك وضح. | مفاهيم/تصورات حول الرعاية التلطيفية المنزلية |
| هل هناك اي شيء اخر تريد ان تشاركه معنا متعلق باستحداث الرعاية التلطيفية المنزلية لمرضى السرطان في غزة؟ | اسئلة أخرى |
