# Supplementary material for: Exploring the perceptions of oncology healthcare professionals on introducing home-based palliative care for patients with advanced cancer in Gaza - a qualitative study
Source: Front Oncol. 2026 Mar 12;16:1786947. doi: 10.3389/fonc.2026.1786947 (PMC13017347; doi:10.3389/fonc.2026.1786947)
Supplement: Supplementary file 2 [file Table2.docx]

**Table 2. Emerging Themes and Subthemes**

| **Main Themes** | **Subthemes** | **Quotes** |
| --- | --- | --- |
| 1- General Perceptions about PC | Acknowledgement of the Palliative Care Definition | *“Palliative care is a comprehensive approach that covers various services including physical, psychosocial and spiritual services.” (*P01*)* |
|  | Misconceptions and Negative Perceptions Towards Palliative Care | *“Patients’ families believe that receiving palliative care indicates that the patient is nearing death. This association creates a negative perception of palliative care.”*  (P01) |
|  | Shifting Perceptions and Overcoming Stigma in Cancer and Palliative Care | *“The more we talk about cancer and palliative care, the more we break the stigma.”* (P01) |
|  | Palliative Care Status in Gaza | *“It is an initiative that I took in 2010 after the death of my father, who died in pain inside a hospital. After this incident, I made the decision that this pain should not be felt by the patient before his death, so I began an initiative to spread the culture of comprehensive pain medicine and palliative care as a new concept in medicine in the Gaza Strip.” (*P01*)* |
| 2- Perceptions of the benefits of HBPC | Benefits to the Patient | *“HBPC would save many patients the hassle of coming to the hospital and waiting in long queues for medications to be dispensed or to be seen by a doctor. These services will reach the patient while he is at home instead.” (*P01*)* |
|  | Benefits to the Healthcare System | *“HBPC frees up hospital beds that can be allocated to other patients in need, optimizing the utilization of healthcare facilities” (*P01*)* |
| 3- Barriers to HBPC Implementation | Geopolitical Context | *“There may be challenges that hinder the implementation of home palliative care, especially in Gaza, because it is a conflict affected area, and it is different from the rest of the world.” (P02)* |
|  | Limited Resources | *“It is possible that there is a ban on some medicines or devices, as it affects the provision of services in the hospital and at home.” (*P03*)*  *"It is difficult to care for the patient at home because essential resources, such as electricity, are often unreliable, whereas hospitals have these facilities available at all times.” (*P04*)*  *“From my point of view, the most prominent challenge is continuity of care because the team dedicated to provide home care is not the same team that works at the hospital. The home team must be free throughout the week and have a day meeting at the hospital to update the care plan for patients in cooperation with the consultant oncologist at the hospital.” (*P05*)* |
|  | Cultural Considerations | *“Gaza being an Eastern society with a Muslim majority, it is considered inappropriate for a male doctor to examine a female patient and vice versa.” (*P03*)*  *“People are accustomed to the hospital being the ideal place for treatment” (*P04) |
| 4- Strategies to Advance Palliative Care in Gaza | Policy Integration, Collaborations, and Research | *“I believe that scientific research is the greatest guide and motivator to convince administration with a specific project.” (*P06) |
|  | Developing a Continuum of Care Model | *"The institution has better resources and can create a structured program for regular patient visits and communication, including a phone app or video chat for patient inquiries, especially useful during conflicts."* (P06) |
|  | Workforce Development and Institutional Support | *“Carrying out training courses specialized in palliative care to develop a medical team specialized in this field, whether by holding internal or other training courses outside Gaza in advanced palliative care centers” (*P07)  *“Awareness first begins with a broad awareness campaign that begins with medical personnel before ordinary people.”* (P03) |
|  | Family and Caregivers’ Engagement | *“Increasing awareness about HBPCs will increase acceptance among patients and their families.”* (P08) |
|  | Cultural Sensitivity and Inclusivity in Palliative Care | *"A holistic approach is vital for meeting patients' needs, especially in a Muslim population, with specialists who can convey ideas of reward and affliction in line with their cultural and religious beliefs."* (P01) |
